# EEG is A Predictor of Neuroimaging Abnormalities in Pediatric Extracorporeal Membrane Oxygenation

**DOI:** 10.3390/jcm9082512

**Published:** 2020-08-04

**Authors:** Jordana Fox, Christopher L. Jenks, Abdelaziz Farhat, Xilong Li, Yulun Liu, Ellen James, Stephanie Karasick, Michael C. Morriss, Deepa Sirsi, Lakshmi Raman

**Affiliations:** 1 Barrow Neurological Institute at Phoenix Children’s Hospital, Phoenix, AZ 85016, USA; jfox1@phoenixchildrens.com; 2 Department of Pediatrics University of Mississippi, Oxford, MS 38677, USA; cjenks@umc.edu; 3 Department of Pediatrics, University of Texas Southwestern Medical Center at Dallas, Dallas, TX 75390, USA; abdelaziz.farhat@utsouthwestern.edu (A.F.); deepa.sirsi@utsouthwestern.edu (D.S.); 4 Children’s Medical Center of Dallas, Dallas, TX 75235, USA; ellen.james@childrens.com (E.J.); Stephanie.Karasick@childrens.com (S.K.); michael.morriss@childrens.com (M.C.M.); 5 Department of Population and Data Sciences, University of Texas Southwestern Medical Center, Dallas, TX 75390, USA; xilong.li@utsouthwestern.edu (X.L.); yulun.liu@utsouthwestern.edu (Y.L.); 6 Department of Radiology, University of Texas Southwestern Medical Center, Dallas, TX 75390, USA; 7 Department of Neurology, University of Texas Southwestern Medical Center, Dallas, TX 75390, USA

**Keywords:** extracorporeal membrane oxygenation (ECMO), electroencephalogram (EEG), neuroimaging, seizures

## Abstract

The goal of this project was to evaluate if severity of electroencephalogram (EEG) during or shortly after being placed on extracorporeal membrane oxygenation (ECMO) would correlate with neuroimaging abnormalities, and if that could be used as an early indicator of neurologic injury. This was a retrospective chart review spanning November 2009 to May 2018. Patients who had an EEG recording during ECMO or within 48 hours after being decannulated (early group) or within 3 months of being on ECMO (late group) were included if they also had ECMO-related neuroimaging. In the early EEG group, severity of the EEG findings of mild, moderate, and severe EEG correlated to mild, moderate, and severe neuroimaging scores. Patients on venoarterial (VA) ECMO were noted to have higher EEG and neuroimaging severity; this was statistically significant. There was no association in the late EEG group to neuroimaging abnormalities. Our study highlights that EEG severity can be an early predictor for neuroimaging abnormalities that can be identified by computed tomography (CT) and or magnetic resonance imaging (MRI). This can provide guidance for both the medical team and families, allowing for a better understanding of overall prognosis.

## 1. Introduction

Extracorporeal membrane oxygenation (ECMO) is increasingly being used as a lifesaving modality in the pediatric intensive care unit. Some of the typical indications include respiratory failure (e.g., asthma, pneumonia, and acute respiratory distress syndrome), cardiac failure (e.g., cardiomyopathy, myocarditis, and arrhythmias), and cardiac arrest. [1].

One of the more devastating complications of ECMO is neurological injury which can occur in up to 35% of ECMO patients. [1,2,3,4] Acute neurologic sequelae (i.e., intracranial hemorrhage, stroke, and seizures) can cause significant morbidity and lead to developmental disabilities in survivors [3]. Studies have shown that neuroimaging severity significantly correlates with a worse neurologic outcome. [5,6,7,8]. Early identification of neurologic injury can potentially help prognosticate the degree of developmental disability and can aid decision making [3]. Due to the problems with transporting ECMO patients, obtaining neuroimaging can be challenging and having a noninvasive, easily repeatable test that could help predict neurologic outcomes during ECMO would be ideal. If a patient still has open fontanelles, a cranial ultrasound with resistive indices may be useful, but cranial ultrasounds are limited to only a subset of the pediatric population [3]. Additionally, they have been known to miss infarctions, small developing hemorrhages, and do not provide an accurate functional assessment of the patient [9].

Electroencephalogram (EEG) is a non-invasive, well-tolerated study that can give dynamic information on cerebral functioning and provide insight into the risk of neurologic sequelae. A 2017 study by Lin et al. looked at a series of 112 patients who received ECMO in which 88% underwent EEG [10]. They found favorable neurologic outcomes in those without seizures versus those with seizures and noted an increased risk of death associated with electrographic seizures [10]. Seizures during ECMO are associated with neurologic complications such as developmental delay, motor deficits, behavioral problems, and decreased quality of life scores [11,12]. Burst suppression and other severe background abnormalities during ECMO are associated with an increased risk of death. [2,10,13].

We hypothesized that the EEG during or shortly after being placed on ECMO would correlate with neuroimaging and could be used as an early indicator of neurologic injury. The first aim of our study was to identify pericannulation patient factors that would correlate with EEG and imaging abnormalities. The second aim of the study was to correlate EEG during or within 48 hours (h) of ECMO discontinuation with neuroimaging completed during or within 3 weeks of ECMO.

## 2. Materials and Methods

### 2.1. Patients

We performed a retrospective analysis to identify pediatric patients who underwent ECMO at the Children’s Medical Center, Dallas, between November 2009 and May 2018, who had an EEG recording and had neuroimaging during ECMO or shortly after being decannulated. The institutional review board (IRB) at the University of Texas Southwestern Medical Center approved the parent study in 2015 and specific to our study in March of 2018. After approval by the institutional review board, we identified patients who met the criteria. The patients were classified as “early” if the EEG was completed during the ECMO run or within 48 hours of decannulation. The patients were classified as “late” if the EEG was completed from 48 hours to 3 months after decannulation. Neuroimaging in the form of computed tomography (CT), magnetic resonance imaging (MRI), and/or head ultrasound (HUS) were obtained on patients within three weeks of ECMO decannulation for the early group and within three months in the late group.

### 2.2. Data Collection

Additional ECMO-related factors were obtained including the ECMO mode, such as venoarterial (VA) or venovenous (VV), site of cannulation, duration of ECMO, presence of ECMO cardiopulmonary resuscitation (eCPR), gender, and age. Laboratory data was obtained for all patients and included the worst serum lactate, lowest pH, highest pCO2, lowest pO2, CBC, BUN/Cr, as well as cannulation site, vasoactive inotrope score (VIS), pediatric risk of mortality score (PRISM), and pediatric logistic organ dysfunction score (PELOD). Due to the wide distribution of the VIS, the VIS was separated into low (less than or equal to 25) and high (greater than 25).

### 2.3. EEG Measurements

EEG was obtained for clinical indication predominantly or as part of post arrest protocol in a few patients. Most common clinical indication was clinical seizures, followed by change in neurological exam or change in vital signs. In the neonatal group indication for EEG were clinical seizures in half of the cohort. EEGs were recorded at the bedside using a portable video machine (Stellate Harmonie, 2009–2011, and Natus Xltek Neuroworks, 2011–2018). Electrodes were applied with paste utilizing the 10–20 international system. A single-lead electrocardiogram was included for all recordings, and neonatal recordings included electrodes to record extraocular movements, respirations, and chin electromyography. All EEG recordings were read and reported by board-certified pediatric epileptologists. EEG scoring was completed from reports and the epileptologist were blinded to the clinical parameters during ECMO and neuroimaging abnormalities. If questions arose regarding reports, raw EEG was reviewed. Tracings were interpreted in accordance with the American Clinical Neurophysiology Society terminology [14]. Electrographic seizures were defined as an acute rhythmic discharge lasting longer than 10 seconds with a temporal-spatial evolution in frequency, amplitude, or morphology. This was termed electro-clinical seizures if the above changes were accompanied by a time-locked clinical change in the patient.

Electroencephalograms included in the study were either long-term (LTM) (greater or equal to 24 h) or routine (30 min–1 h) recordings. EEG recordings were sorted by type (long-term or routine), age of the patient at the time of the study (neonatal (<44 weeks corrected gestational age) or pediatric, and severity score (mild, moderate, or severe). Gestational age was taken into account for the neonatal patients. The EEG severity score was determined for early and late EEGs. For the early group, patients typically had only one EEG study (although some were over the course of multiple days or long-term monitoring EEG (LTM)). For the late group, we only used the EEG closest to ECMO. Scoring was developed for the neonatal group and childhood group, with mild, moderate or severe EEG changes. Scoring for neonates was adapted from a previously used system from 1983, by Thorp and Laboyrie [15]. Childhood scores were developed based on similar principles and also scored based on the severity of background abnormalities and seizures. Refer to Table 1 and Table 2 for details of EEG scoring.

### 2.4. Neuroimaging Assessments, Scoring and Classification

Neuroimaging scoring was performed by an independent neuro-radiologist who was blinded to EEG and ECMO related data. The neuroimaging was reviewed on each patient and included in the ultimate score. If the patient had more than one modality of neuroimaging, CT imaging during ECMO if available was used; post ECMO MRI was used over HUS when available and HUS was used only when neither CT nor MRI images were unavailable. Scoring was adapted from Taylor et al., 1987, with a scoring guide producing a weighted score based on the category of ventricular dilatation, bleeding, and parenchymal lesions [6]. A score of 0–4 was considered mild, 5–9 was considered moderate, and greater than 10 was considered severe. A total of 10 images from the early EEG group cohort were reviewed independently by a second neuro-radiologist who was also blinded to the EEG and ECMO related data. The scores assigned by both radiologists were then reviewed for concordance. Cohen’s Kappa statistic was 0.82, indicating near perfect agreement between the reviewers.

### 2.5. Statistical Methods

Descriptive information for continuous variables was presented by mean ±SD based on distribution, and categorical variables were presented as percentages. Comparison between groups was performed by the Chi-square test for categorical variables, and the *t*-test for Gaussian distributed continuous variables. The Wilcoxon rank sums test was applied for the non-Gaussian distributed data, to compare continuous severe neuroimaging scores among three EEG levels: mild, moderate, and severe EEG. The trend analysis by Jonckheere-Terpstra test showed the more severe the EEG levels, the higher the neuroimaging score. All analyses were performed using SAS 9.4 (Cary, NC, USA).

## 3. Results

### 3.1. Patients

**Figure 1:**
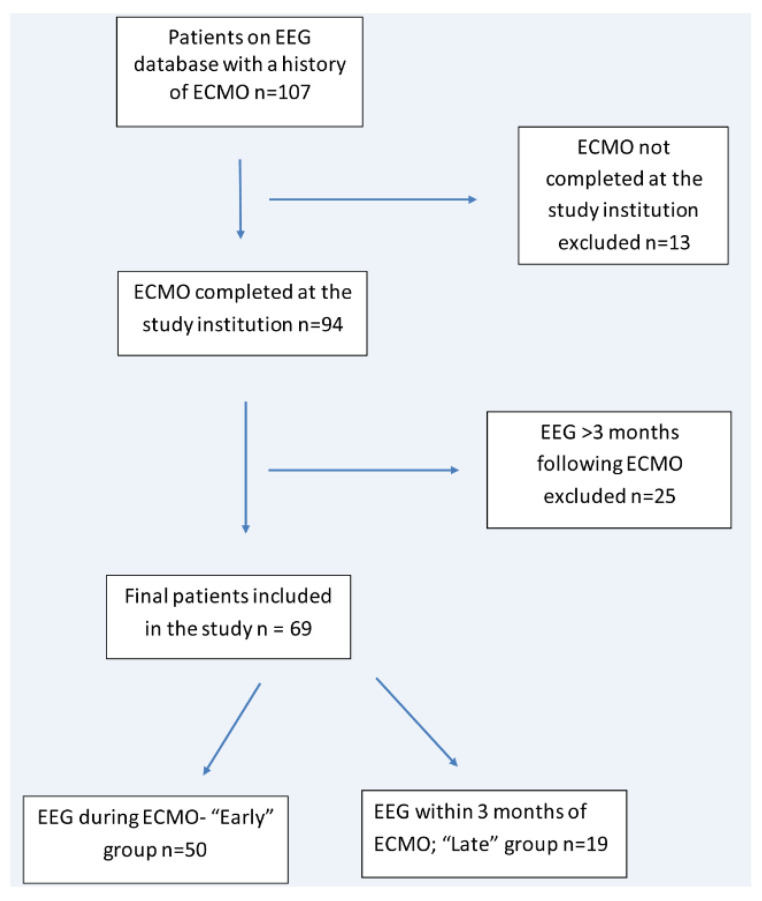
Schematic outlining inclusion and exclusion of patients in the study. Extracorporeal Membrane Oxygenation (ECMO), Electroencephalogram (EEG).

A total of 69 unique patients met the inclusion criteria. Initially, 107 patients were identified from our EEG database with a history of ECMO. After excluding patients who did not receive ECMO at our institution and who did not have EEG either during ECMO (early group) or within 3 months of their ECMO course (late group), we arrived at the total number of 69 patients. A total of 50 were in the early group, and 19 were in the late group (Figure 1).

### 3.2. Early Group

In the early group, 44 patients had an EEG performed during ECMO, and 6 patients were off ECMO for 24–48 h prior to the EEG. Within the early group, there were 29 males, 18 were neonates, and 32 were children. Of these patients, 39 were VA ECMO, and 11 were VV ECMO. Predominant diagnosis was ARDS, sepsis and underlying cardiac disease (Demographics Table 3). We did not identify any pericannulation factors to be significant. There was no statistical significance in comparing the severity of EEG with any of the lab values that we collected (data not shown) and in particular the VIS (*p* = 0.96) or the serum lactate (*p* = 0.23). Overall survival was around 65% across all EEG categories.

### 3.3. EEG and Neuroimaging Findings

**Figure 2:**
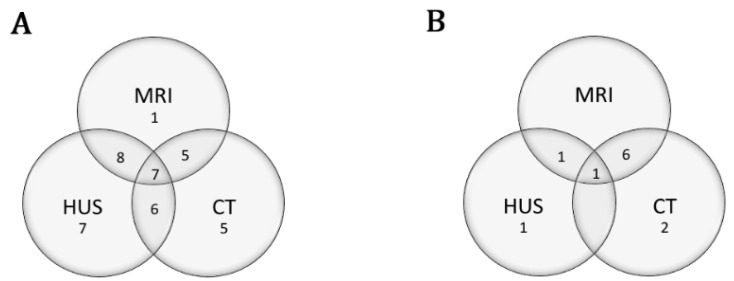
Neuroimaging types for patients in early group. Areas of intersection correspond with patients who had multiple neuroimaging modalities. The number represented in each area represents the number of patients (*n*) who received each type of the adjacent neuroimaging. (**A**) Neuroimaging in patients who received VA ECMO, *n* = 39 (**B**) Neuroimaging in patients who received VV ECMO, *n* = 11 VA: veno-arterial; VV: veno-venous; ECMO: extracorporeal membrane oxygenation. MRI: magnetic resonance imaging, HUS: head ultrasound, CT: computerized tomography.

In the VA ECMO group, there was 1 patient with only MRI, 5 with only Head CT, and 7 with only HUS. The remaining 26 patients had a combination of neuroimaging with at least two of the three imaging studies (Figure 2A). In the VV ECMO group, there was 1 patient with only HUS, 2 with only Head CT and the remaining 8 patients had a combination of neuroimaging (Figure 2B).

**Figure 3:**
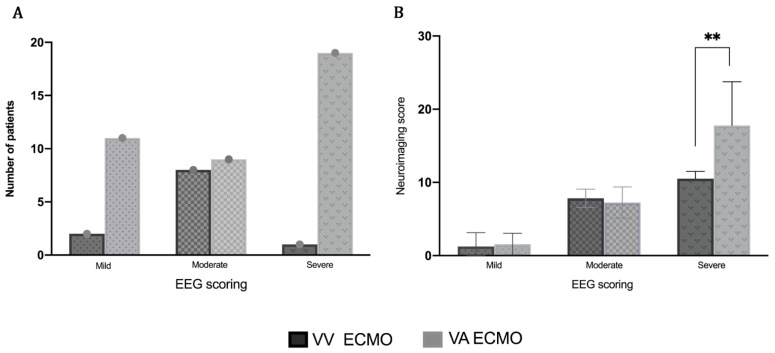
Severity of EEG scores and neuroimaging scores for VA and VV ECMO group patients. The EEG severity is noted across the x-axis in both graphs. (**A**): The number of patients is on the y-axis. VV ECMO is demonstrated in dark gray and VA ECMO in light gray. Patients on VA ECMO show a higher severity of EEG scores (*p* = 0.007). (**B**): The neuroimaging severity score is on the y-axis. VV ECMO is demonstrated in dark gray and VA ECMO is in light gray. Patients showed a statistically significant progression in the severity of their EEG score in relation to their neuroimaging score severity (*p* < 0.0001). EEG: electroencephalogram; VA: veno-arterial; VV: veno-venous; ECMO.

In the VA ECMO group, a total of 19 were LTM and 20 were routine EEGs. In the VV ECMO group, a total of 4 were LTM and 7 were routine EEGs. Overall, 13 patients had mild EEG findings, 17 had moderate EEG findings, and 20 had severe EEG findings. The difference in EEG severity in the VA versus the VV ECMO patients was statistically significant (*p* = 0.007) (Figure 3A). In the VA group, there were 11 with mild EEG severity, 9 with moderate EEG severity and 19 patients with a severe EEG score. In the VV group 2 patients had mild EEG severity, 8 with moderate EEG severity and 1 had a severe EEG score. Overall, neuroimaging revealed 19 patients with mild neuroimaging scores, 9 patients had moderate neuroimaging scores and 22 had severe neuroimaging scores (Figure 3B). In the VA group, 15 had mild neuroimaging severity, 6 had moderate neuroimaging severity and 18 had severe neuroimaging scores severity. In the VV group, there were 4 mild neuroimaging scores, 3 moderate neuroimaging scores and 4 that had severe neuroimaging scores.

**Figure 4:**
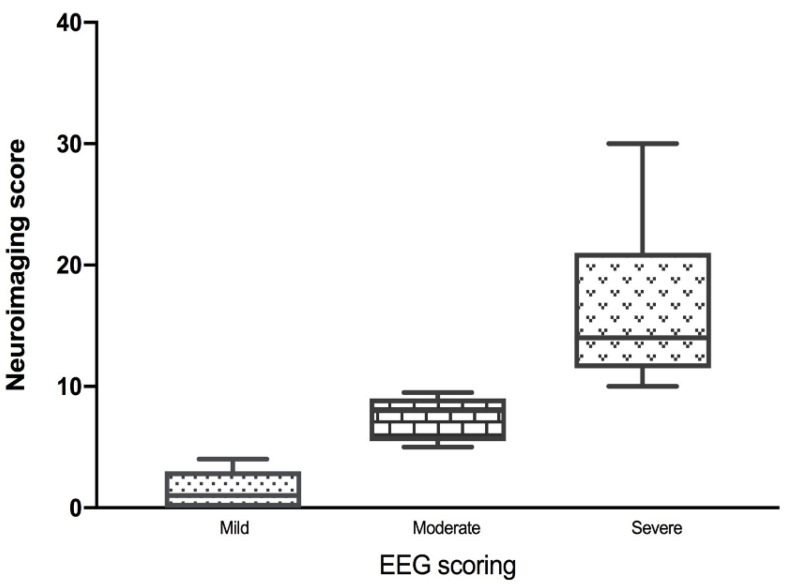
EEG severity corresponds with neuroimaging severity. Representative of all early patients, EEG severity score is along the x-axis and neuroimaging severity score is along the y-axis. A box plot representation of mild, moderate, and severe EEG scores corresponding with mild, moderate, and severe neuroimaging scores (*p* < 0.0001). EEG: electroencephalogram.

There was a statistically significant difference when comparing the mild, moderate, and severe EEG findings to mild, moderate, and severe neuroimaging scores (*p* < 0.0002) (Figure 4). The trend analysis by Jonckheere-Terpstra test showed the more severe of EEG levels, the higher of neuroimaging score with a trend p value of <0.0001 (Figure 4). In a further breakdown of the severe EEG group into those with a severe background abnormality but no seizures (9/20), those with seizures but not abnormal background (2/20), and those with both an abnormal background and seizures (10/20), there was no statistical significance (*p* = 0.1685) when compared to neuroimaging scores (*n* = 20).

### 3.4. Medications

In the early group who underwent EEG during the acute ECMO period, multiple medications were utilized for sedation. Of the 50 patients, 47 were treated with an opioid (fentanyl, morphine, hydromorphone, or methadone). There were 33 patients who were treated with a benzodiazepine (midazolam, diazepam, or lorazepam), of which the majority were on midazolam infusion (32/33). Doses of midazolam were between 0.025 mg/kg/h to 2 mg/kg/h, with a majority (23/33) treated with doses at or below 0.3 mg/kg/h. A total of 12 patients were treated with an α-2 agonist (dexmedetomidine, +/− clonidine). Doses of dexmedetomidine were between 0.2mcg/kg/hour and 1.5 mcg/kg/h. Of these 12 patients, 6 received doses at or less than 0.5 mcg/kg/h. Two patients were on a pentobarbital drip at a maximum of 2 mg/kg/h. One patient received pentobarbital infusion for treatment of increased intracranial pressure (ICP) from large right middle cerebral artery stroke. A second patient also received pentobarbital infusion for increased ICP secondary to traumatic brain injury from motor vehicle accident. Both patients had EEGs that were categorized as moderate severity and no findings of burst suppression or more severe changes were reported. There were 26 patients of the 50 who received anti-seizure medications (ASM) bolus or scheduled doses during the time of EEG recording. A total of 18 patients received one ASM, 5 received 2 ASM, and 3 received 3 ASM. Medications used included levetiracetam, phenobarbital, lacosamide, valproic acid, and fosphenytoin. Levetiracetam and phenobarbital were the most common medications used.

### 3.5. Late Group

In the late group, EEG was obtained within one week in eight patients, one month in seven patients and greater than one month but less than 3 months in six patients, following ECMO decannulation. Within the late group, there were 11 males, 8 females (2 females with 2 ECMO runs), 9 neonates (1 neonate with 2 ECMO runs), and 10 were children (1 child with 2 ECMO runs). Three patients had only CT scans, five patients had only HUS, and one patient had only an MRI. The remaining twelve patients had a combination of at least two of the three studies. Five patients had a normal EEG. Five patients had mild, three had moderate, and eight had severe EEG abnormalities. In the late group, there was no statistically significant difference between those with mild, moderate or severe EEG findings in relation to the severity of neuroimaging scores (*p* = 0.54).

## 4. Discussion

In our single center study, seizures were identified in 24% patients, and the severity of early EEG scores corresponded with severity of neuroimaging scores. Severity of EEG and neuroimaging was much higher in the VA ECMO group.

The severity of EEG abnormalities during ECMO that corresponded with severity of neuroimaging abnormalities has been partly supported in recent studies where seizures during EEG were linked to brain hemorrhage and brain injury [2,16,17]. A prior study where only a combination of HUS and EEG was highly predictive of abnormal neuroimaging findings may be secondary to the use of routine EEG, as opposed to LTM, lack of scoring system for neuroimaging abnormalities, and proximity of neuroimaging to the ECMO run [18]. A 2015 systematic review of the literature on neuromonitoring methods during ECMO reported that most studies found no correlation between abnormal EEGs and HUS, CT Head, or MRI brain findings during or after ECMO [19]. It is essential to recognize that utilization of more sensitive neuroimaging such as MRI and longer-term EEG studies, such as LTM, may better detect underlying neurologic sequelae.

Our study also noted a graded association between EEG and MRI findings, that is mild and severe EEG findings corresponded with similar imaging findings, which suggests that EEG during ECMO may bear predictive potential for MRI abnormalities and can aid prognosis. Additionally, we were able to provide further reassurance that mild EEG findings corresponded with only mild neuroimaging findings. The inclusion of other severe EEG abnormalities, such as voltage attenuation of the background and burst suppression in the severe EEG group, in addition to seizures, may account for the association with severe imaging abnormalities. Though the significance of background EEG abnormalities have been described by Lin et al., this finding along with seizures, needs further exploration in future studies to establish their association with neuroimaging abnormalities and predictive potential in ECMO patients [10]. In addition, concurrent medications that may influence background EEG features will have to be assessed in detail. Future prospective studies which use long-term EEG monitoring with other neuromonitoring modalities to assess cerebral blood flow as standard care for all ECMO patients may help correlate findings between these modalities and neurodevelopmental outcome. In addition, this may lead to the detection of early brain injury markers and the development of better neuroprotective strategies to improve outcomes.

An association of VA causing more neurological injuries compared to VV ECMO has been found in the ELSO registry data [20,21]. We found a similar correlation with higher neuroimaging and EEG scores in children treated with VA ECMO. Cannulation of the major vessels has a greater risk of ischemic and thromboembolic events which may account for higher neurologic injuries with VA ECMO [22,23]. However, single-center studies in both neonatal and pediatric patients have not found differences between VA, VV ECMO, and occurrence of seizures during EEG [16]. Our results may have been influenced by the fact that more than 70% of our patients were on VA ECMO. 

In our study seizures occurred in 12 of the 50 patients (24%) which is similar to other studies. Three recent studies using continuous EEG monitoring have demonstrated that the risk for seizures in ECMO patients is substantial, with seizures detected in 18%–23% of children and 50%–83% of those having electrographic only seizures [2,16,24]. Our study further supports the indication for long-term EEG monitoring of these patients due to the high risk for electrographic only seizures.

There was no statistically significant association between neuroimaging and EEG severity scores in our late EEG group. There are several explanations for this; however, the most significant was likely the low number of patients within this group, with only 21 total ECMO runs, in 19 distinct patients, identified. In addition, having EEG during ECMO may provide the key to acute dynamic changes occurring within the brain. In contrast, later EEG evaluation has allowed those dynamic changes to become static and less distinguishable during chronic stages and recovery. This is especially true in the neonatal and younger pediatric population, where neuroplasticity is high [25,26]

There were no clear associations made between type, the dose of sedative medications, and the EEG findings. The analysis was highly limited by the variable duration of EEG, variable types, and dosages of sedative medications. Sedative medications such as opiates and benzodiazepines can affect the EEG. Opiates can have mild effects on EEG, with alpha rhythm slowing. Benzodiazepines can have mild to moderate effects on EEG with alpha rhythm slowing, increased theta, increased beta activity, and decreased amplitudes. Pentobarbital infusions are used during refractory status epilepticus and for reduction of intracranial pressure and dose is titrated to achieve and maintain EEG pattern of burst-suppression [27,28]. A majority (94%) of our patients were on an opiate infusion, and around two thirds (66%) of patients received benzodiazepine infusions during the EEG. Two patients received pentobarbital for increased ICP but neither patient was titrated to burst suppression and their EEGs were scored in the moderate category. A total of 52% of patients received an ASM during the EEG, with the majority only receiving one ASM (70%). Although statistical analysis could not be performed, we did not observe a direct association between medication types, doses, and EEG severity.

### Limitations

There were several limitations to this study. The selection of patients who underwent EEG may have had bias for more severe degrees of injury. Additionally, the differing timing and duration of both the EEG monitoring and neuroimaging, as well as the impact of concurrent medications on EEG findings, were limitations. The study was over a 10-year time period, over which significant advances in ECMO technology and neuroimaging have taken place, which could influence some of our results.

## 5. Conclusions

Neurologic injury during ECMO is associated with significant morbidity. EEG monitoring during ECMO can help identify patients at risk for neurologic injury. Our study highlights that EEG severity can be an early predictor for neuroimaging abnormalities and adds to other recent studies that have informed us on the importance of long-term EEG in identifying risk of neurologic sequelae in children who receive ECMO. This can provide guidance for both the medical team and families, allowing for a better understanding of overall prognosis. Further prospective multicenter studies with standard EEG monitoring with controlled variables may be a key to determining if EEG can serve as an early marker of neurologic injury.

## Figures and Tables

**Table 1:** Neonatal Electroencephalogram (EEG) severity scoring (adapted from Tharp and Laboyrie, permissions obtained).

Normal	Mild EEG	Moderate EEG	Severe EEG
	Inconsistent Interhemispheric Asymmetry Mild Focal Abnormalities Mild Disturbance in Background (Paucity of Theta, Excessive Sharp Transients) Mild Excessive Inter Hemispheric Asynchrony Mild Excessive Discontinuity Increase in Number of Frontal Sharp Waves	Excessive Discontinuity of Asynchrony Moderate Persistent Asymmetry of The Background Focal Sharp Waves, Spikes or Delta Activity in Addition to Other Abnormalities Dysmaturity Occasional Positive Rolandic Sharp Waves with Abnormal Background Excessive Diffuse Background Delta Activity with Excessive Discontinuity	Isoelectric Marked Increased Inter Burst Intervals Paroxysmal Background with Or Without Excessive Inter Hemispheric Asynchrony Low Voltage (<20 uV), Diffusely Slow Background Electrographic or Electroclinical Seizures Abundant Positive Rolandic Sharp Waves

**Table 2:** Childhood EEG severity scoring.

Normal	Mild EEG	Moderate EEG	Severe EEG
	Intermittent Generalized Slowing Continuous Reactive Generalized Slowing Mild/Intermittent Focal Slowing Mild/Intermittent Focal Asymmetry	Continuous Non-Reactive Generalized Slowing Interictal Epileptiform Discharges Lateralized Periodic Discharges Continuous Focal Asymmetry	Electrocerebral Inactivity Burst-Suppression Very Low Voltage Activity (<20 uV) Electrographic or Electroclinical Seizures (Including Status Epilepticus)

**Table 3:** Demographic characteristics of patients in the mild, moderate, and severe EEG groups.

Patient Group, *n* (%)	Mild EEG, 13 (26%)	Moderate EEG, 17 (34%)	Severe EEG, 20 (40%)
Age Group, *n* (%)			
Neonates (<44 weeks CGA)	6 (46%)	4 (24%)	8 (40%)
Children	7 (54%)	13 (76%)	12 (60%)
Gender, *n* (%)			
Male	9 (69%)	12 (71%)	8 (40%)
Female	4 (31%)	5 (29%)	12 (60%)
Primary Diagnosis, *n* (%)			
PPHN	0 (0%)	0 (0%)	1 (5%)
Sepsis	0 (0%)	3 (17.6%)	1 (5%)
ARDS	3 (23%)	5 (29.4%)	1 (5%)
Cardiac	2 (15.4%)	1 (5.9%)	6 (30%)
ECMO Type, *n* (%)			
Veno-Arterial	11 (85%)	9 (53%)	19 (95%)
Veno-Venous	2 (15%)	8 (47%)	1 (5%)
Site of ECMO Cannulation *n* (%)			
Neck	11 (85%)	12 (71%)	16 (80%)
Femoral	0 (0%)	1 (6%)	0 (0%)
Central	2 (15%)	4 (23%)	4 (20%)
Duration of ECMO, h, mean +/− SD	807.4 +/− 667.5	669.3 +/− 398.5	230 +/− 180.7
Pediatric Risk of Mortality Score (PRISM), Mean +/− SD	27.6 +/− 5.6	30.9 +/− 4.4	31.3 +/− 3.7
Pediatric Logistic Organ Dysfunction Score (PELOD), mean +/− SD	15.1 +/− 10.8	14.9 +/− 9.3	22.6 +/− 16.3
Presence of ECMO CPR (eCPR), *n* (%)	3 (21.4%)	4 (28.6%)	7 (50%)
Neuroimaging Severity Score, *n* (%)			
Mild	10 (77%)	5 (29.5%)	4 (20%)
Moderate	3 (23%)	5 (29.5%)	1 (5%)
Severe	0 (0%)	7 (41%)	15 (75%)
Survival, *n* (%)	9 (69.2%)	11 (64.7%)	13 (65%)

PPHN: Persistent pulmonary hypertension, ARDS: Acute respiratory distress syndrome, ECMO: Extracorporeal Membrane Oxygenation.
